# Microbial contamination of keyboards and electronic equipment of ICU (Intensive Care Units) in *Kashan University of medical sciences and health service hospitals*

**DOI:** 10.1016/j.mex.2019.03.022

**Published:** 2019-03-28

**Authors:** Mehdi Nazeri, Javad Salmani Arani, Narjes Ziloochi, Hasan Delkhah, Mohsen Hesami Arani, Esrafil Asgari, Mona Hosseini

**Affiliations:** a Department of Parasitology, School of Medicine, Kashan University of Medical Sciences, Kashan, Iran; b Department of Environmental Health Engineering, Deputy of Health, Kashan University of Medical Sciences, Kashan, Iran; c Department of Environmental Health Engineering, School of Public Health, Iran University of Medical Sciences, Tehran, Iran; d Department of Environmental Health Engineering, Khoy University of Medical Sciences, Khoy, Iran; e Department of Environmental Health Engineering, School of Health, Isfahan University of Medical Sciences, Isfahan, Iran

**Keywords:** Microbial contamination of keyboards and electronic equipment, Contamination, Keyboards, Electronic equipment

## Abstract

Microbial contamination of computer keyboards and inanimate surfaces of electronic equipment in ICU (Intensive Care Units) can have a significant role for ICU-acquired colonization and a spectrum of nosocomial infections. The aim of this study was to survey the incidence of bacterial contamination and the distribution of species of computer keyboards and inanimate surfaces of bed side equipment in ICUs in Kashan University of medical sciences and health service hospitals. This descriptive, cross-sectional study was done on 75 computer keyboards and inanimate surfaces electronic equipment in 5 ICUs during 2016–2017. Samples were collected from computer keyboards and electronic equipment with normal saline rinsed swabs. Samples were Cultivated on Blood Agar (BA), and MacConkey Agar (MAC) and growing bacteria were identified based on their morphology and biochemical properties. Seventy six (76%) out of 75 computer Keyboards and electronic equipment were contaminated with bacteria and fungi. The most contamination pertained to gram positive bacteria (70.7%) and the most isolated bacteria were coagulase-negative *staphylococci*. The highest contamination rates were found on computer keyboards and electronic equipment of which were nurses.

• This study demonstrates that monitoring inanimate surfaces and considering these surfaces as source of nosocomial infections is necessary.
• In total, Seventy six (76%) out of 75 computer keyboards and electronic equipment in ICUs had positive culture.
• It can be concluded that it is necessary for ICUs of Kashan university of medical sciences and Health service hospitals to have practical and regular program to reduce nosocomial infections.

## Graphical abstract

**Figure:**
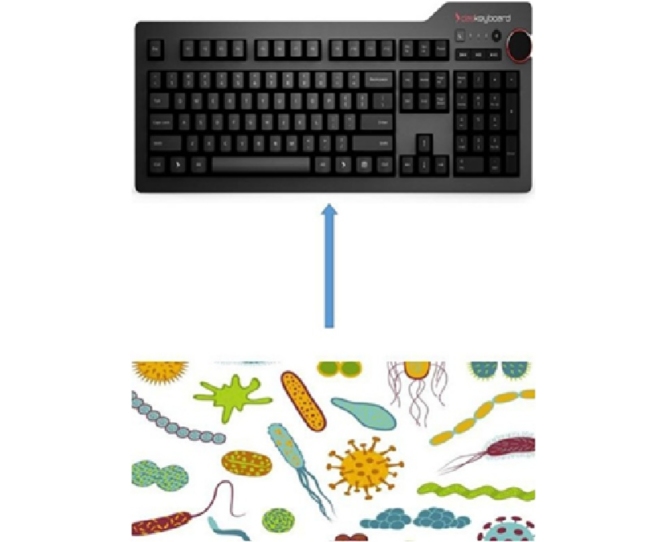

Specifications Table

**Table:** 

Subject area:	Environmental Science
More specific subject area:	Microbial contamination
Methods name:	Microbial contamination of Keyboards and electronic equipment
Name and reference of original method:	Nepomuceno DB, Lima DV, Silva MO, Porto JCS, Mobin M. Evaluation of disinfectants in order to eliminate fungal contamination from computer keyboards of an integrated health center in Piauí, Brazil. Environmental monitoring and assessment. 2018;190(10):608
Resource availability:	The data are available with this article

## Method details

Nowadays, health care infections and the associated high mortality rate is very high in many hospitals [1]. Nosocomial infections are one of the most important infections, that occur in patients hospitalized due to non-infectious diseases and are considered as one of the main causes of mortality in hospitals, which subsequently increase the cost of health cares for the health system [[2], [3], [4], [5], [6]]. Recently, nosocomial infections are considered as the most important causes of mortality and morbidity among patients admitted in to Intensive Care Unit (ICU) while the majority of patients admitted in to these wards are immunocompromised. In addition, the bacterial agents of this diseases are multidrug-resistant (MDR) that could cause many problems for health care facilities and enormous challenges for physician and hospital administrators around the world [[7], [8], [9], [10], [11]]. The worldwide prevalence of nosocomial infections are estimated near 10% while in Iran it has been reported between 1.3–10% [[12], [13], [14], [15], [16], [17]]. Only in the United States, more than two million nosocomial infections were reported which cost more than 4.5$ million and has resulted in 90,000 deaths annually [18]. Medical staffs and nurses are supposed to be a common mode of exogenous transmission through their hands. The role of the hospital environment as a reservoir of nosocomial pathogens has been controversial [19]. However, electronic equipment and computers at the bedside of patients in health service hospitals can contribute to the transmission of pathogens in hospital infections [20,21].

In different studies, keyboards are known as the reservoirs of pathogens, because they are used at the rooms where the patients are especially because of repeated contacts with the hands of the personnel [1,19,22].

Poor hygiene of hands of health professionals and the ubiquity of computer accessories in hospital environments, caused keyboard and mouse known as a potential source of cross-infection in these environments also microorganisms transferred from surfaces of computer accessories to the bare or gloved hands of health care staffs [23,24].

Patients with a critical diseases in ICUs seem to need more nursing care, and as a consequence the chance of cross-transmission of microorganisms by the hand of care personnel from these fomites are assumed as additional risk factor for acquiring nosocomial infections [19].

Several studies have demonstrated the microbial contamination of computer equipment. A study was conducted by William A. Rutala et al., they reported potential pathogens including staphylococci, diphtheroids, Micrococcus species, and Bacillus species in more than 50% of the computers [14,25].

Studies were conducted in Iran showed microbial contamination exists on the all keyboards of hospitals [15,22]. In this regard, current study was conducted to assess the incidence of bacterial contamination and the distribution of species on computer keyboards and bed side equipment in the ICUs of Kashan hospitals, where the patients were immunocompromised.

## Material and methods

This descriptive, cross-sectional study was conducted on 75 computer keyboards and equipment in 5 ICUs (intensive care units) of Kashan hospitals. This study focuses on keyboards and equipment of ICU specifically, with a more numbers compared to similar studies in Iran.

At first, all information related to computer keyboards and electronic equipment such as location, type and number of users (medical or non-medical staff), disinfection method and disinfectants were recorded. Computer keyboards and electronic equipment were randomly tested during routine daily activity of users.

Then sampling was done by sterile swab soaked in normal saline) along side the alcoholic lamp). The sampling method was such that swab rolling over of the surfaces of the mostly used keys (Enter, Space bar, P, D, K keys), that having area near to 16 cm^2^. As well as, touch electronic keys of other equipment were sampled from area similar to computer keyboards (16 cm^2^) which was broader than similar studies, also surfaces sampled randomly.

After sampling, swabs were inserted into the test tubes containing the culture medium (Tryptic Soy Broth) and was transferred to the lab in the shortest time.

Contaminated swabs were streaked on Blood and MacConkey Agar (Himedia, India). All agar plates were incubated for at least 48 h at 37 °C. All positive cultures identified based on conventional microbial diagnostic methods as gram staining, colony morphology, color and the presence or absence of hemolysis. Gram positive bacteria were identified by applying catalase and coagulase tests, sensitivity to novobisin (staphylococci), and bile sculin selective medium (enterococci).

Also gram-negative bacteria were discriminated with oxidase and IMVIC tests which were useful for differentiation of Enterobacteriaceae [26,27]. All data was analyzed by Chi 2 statistics tests using statistical software (SPSS 16).

## Results

In total, fifty-seven (76%) out of 75 computer keyboards and electronic equipment in ICUs had positive culture. As the results demonstrate the highest positive cultures belonged to gram positive bacteria (70.7%). Coagulase negative bacteria (72%) were the most frequent isolated from 75 samples taken from computer keyboards and electronic equipment followed by micrococcus (48%), gram positive bacillus (18.6%), viridans streptococci (9.3%), nonfermenting gram-negative bacilli (9.3%), Enterobacteriaceae (12%), staphylococcus aureus (6.6%), non-spore gram positive bacillus (4%) and enterococcus (3.5%). Moreover, among all 75 samples, 38.6% and 18.6% were contaminated to saprophytic fungal hyphae and yeast, respectively (Table 1).

**Table 1:** Frequency of organisms isolated from computer keyboards and electronic equipment of ICU Units in Kashan university of Medical sciences and hospitals.

Microorganism	Positive		CFU	
	Sample	%	Mean	Median
*Staphylococcus aureus*	5	6.7	18	12
Coagulase-negative *staphylococci* spp.	54	72	14	10
*Enterococcus* spp.	2	3.5	22	–
*Micrococcus* spp.	3			
Viridans *streptococcus* spp.	7	9.3	5	3
Bacillus spp.	14	38.6	19	14
Nonfermenting gram-negative bacilli spp.	7	9.3	16	12
*Enterobacteriaceae* spp.	9	12	4	3
Yeast spp.	29	38.6	6	3

Contamination rates of computer keyboards and electronic equipment in patients, room and work places were 83.7% and 64.5%, respectively. Despite the higher level of contamination of electronic equipment in patient rooms, this difference was not statistically significant (p = 0.138).

Among all contaminated keyboards and electronic equipment, 73.7% and 84.6% had cleaning program once a day and once a week so that this difference was not statistically significant (p = 0.6).

**Fig. 1:**
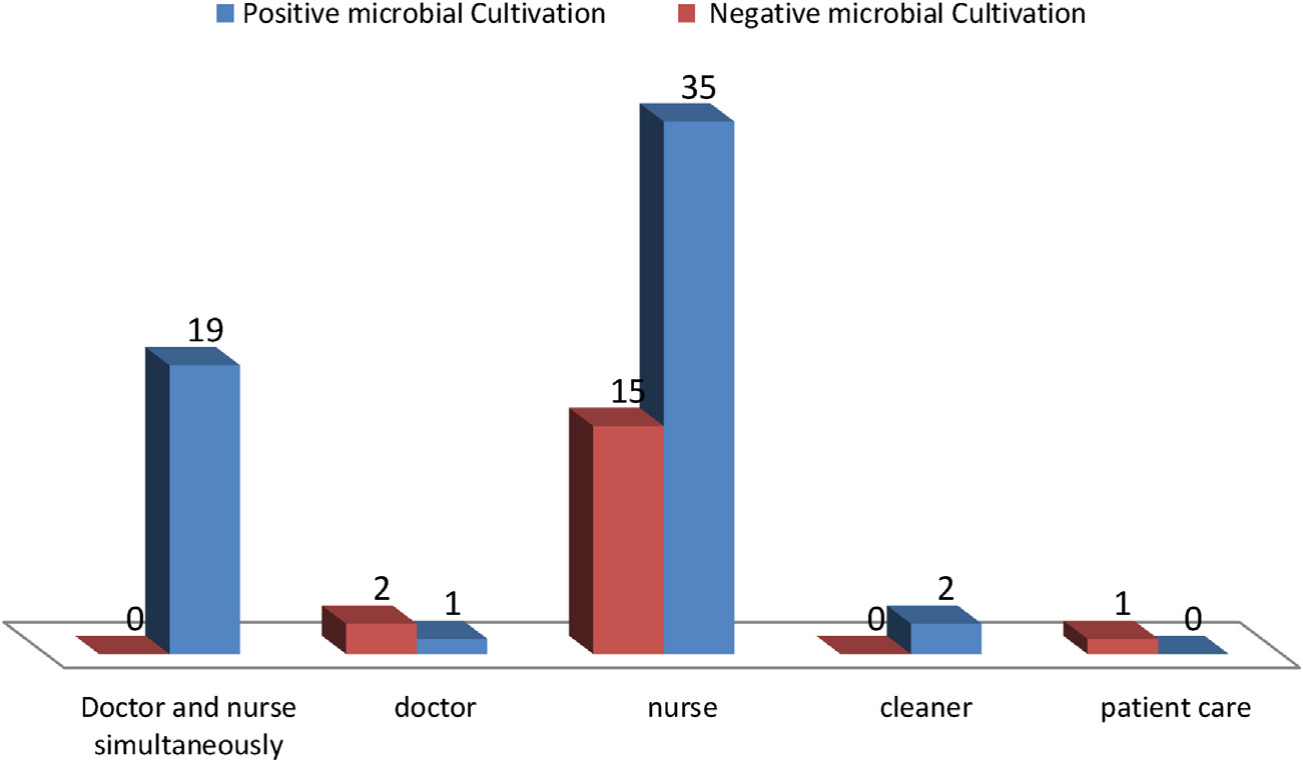
Comparison of Positive and Negative microbial Cultivation of Computer Keyboards and Electronic Equipment based on Type of Users in ICU Units in Kashan university of Medical sciences and hospitals.

The most users of the computer keyboards and electronic equipment were nurses or doctors and nurses simultaneously and contamination rates for them were 100% and 70%, respectively which was statically significant (Fig. 1). Saysept, Ethanol and D2 Disinfecting Spray were used as disinfectants.

## Discussion

In this study more than two thirds (76%) of all computer keyboards and electronic equipment in ICUs were infected with microorganisms that could contribute to the development of hospital infections. However, most of them were arranged as closely as possible to the bed of patients so that the chances of cross-transmission through health care personnel were more than expected. Patients in ICUs suffer from numerous predisposing factors for leading to nosocomial infections such as central venous catheter, total parenteral nutrition, prolonged and broad-spectrum antibiotic therapy, previous steroid or immunosuppressive therapy, repeated abdominal surgeries and low birthweight [9].

The potentially pathogenic microorganism could be found on plastics surface as computer keyboards and other fomite surface so these could act as a reservoir for nosocomial infections [12]. Also, the common transmitter of exogenous nosocomial infections is the hand of health care staffs. Therefore, computer keyboards and other fomite in patient rooms have an important role in acquired nosocomial infections via staff hands [13,14].

However, due to excessive contact with computer keyboards, electronic equipment and other fomites surface and the carelessness of personnel and patients has led to a reservoir and source for nosocomial infections. This process eventually results in lower compliance with hand hygienic regimens among doctors and nurses [15]. Hartmann et al. in their research on microbial contamination of computer keyboards, mouse and other fomites in a surgical ICU of a tertiary teaching hospital, concluded that the colonization rate in the keyboards and mouse was higher than on other (non-porous) surfaces [9]. So that, in one study under the experimental conditions, the highest bacterial transmission from surfaces to the hands was from smooth surfaces such as water taps and telephone receivers [14].

In our study, the contamination rate (76%) was lower than some other researches. However, in some studies it was reported up to 100% [14,16,18].

Different studies reflected a diverse contamination rate based on type of bacteria, but in this report the highest rate of contamination was due to coagulase-negative *staphylococci* [13,14,18,19]. In one another study all computer keyboards were contaminated with *enterobacteriacea* which this disagreement were justified by difference in method of sampling and disinfecting [19].

The contamination rate among different rooms (nurses’ station, patient room) was not statistically different which is similar to Engelhart,s view stating that increasing contamination for varied situation and location is unpredictable [19]. In another investigation, Bures et al. reported an unvarying contamination rate in internal medicine ICU without being affected by proximity to patients or the geographic position within the ward [21]. Although in this study staffs hand flora were not investigated, other study demonstrated that both medical care staffs and non- medical staffs could play as a reservoir for nosocomial pathogenic bacteria. However, non-medical staffs isolated bacteria were clinically more important and had a higher resistance pattern [17,21].

Russotto et al. reported the colonization and contamination of inanimate surfaces may have a significant role in microorganism cross-transmission in ICU. Health care staff hands and shedding of bacteria from hand on inanimate surface is the main route of contamination in ICUs. Also, environmental contamination was different in infected patients and in those admitted for non-infectious reasons. Moreover, assumed infection occur not only through direct contamination but also after touching bacteria on inanimate surfaces which are isolated from patients and have the same susceptibility profiles of those isolated from some equipment in ICU [17].

*Enterococci* were the least isolated bacteria. However, since *enterococci* is considered as an indicator of fecal contamination. Therefore any isolation, regardless of its number, is a valuable in environmental health aspect [25].

However, the pattern of isolated bacteria could be affected by disinfection protocols and considered to hygienic health programs which are used by health care staffs. In this study, 38.6% of the computer keyboards and electronic equipment were contaminated to hyphal fungi, having the highest frequency associated with Aspergillus flavus, Chaldesporium and Penicillium. This frequency was near to distribution fungal specie in selected ward in Imam Khomeini Hospital, and children's medical center, in Tehran, Iran [28]. However, the researchers concluded that, this frequency was related to the fungi isolated from computer keyboards and electronic equipment.

## Conclusions

This study demonstrates that monitoring inanimate surfaces and considering these surfaces as source of nosocomial infections is necessary. We suggested practical and regular program to reduce nosocomial infections in ICUs of “Kashan university of medical sciences and hospitals” comprising: application of effective disinfectant, methods of regular and appropriate disinfection of keywords surfaces, and appropriate education.

## Conflict of interest

The authors of this article declare that they have no conflict of interests.
